# Proton Pump Inhibitor for Gastrointestinal Bleeding in Patients with Myocardial Infarction on Dual-Antiplatelet Therapy: A Nationwide Cohort Study

**DOI:** 10.1007/s44197-024-00267-9

**Published:** 2024-06-24

**Authors:** Minyoul Baik, Jimin Jeon, Jinkwon Kim, Joonsang Yoo

**Affiliations:** Department of Neurology, Yongin Severance Hospital, Yonsei University College of Medicine, 363, Dongbaekjukjeon-daero, Yongin-si, 16995 Gyeonggi-do South Korea

**Keywords:** Dual-antiplatelet therapy, Myocardial infarction, Proton pump inhibitor, Gastrointestinal bleeding

## Abstract

**Background:**

Guidelines provide various recommendations for the use of proton pump inhibitors (PPI) to prevent upper gastrointestinal (UGI) bleeding in acute myocardial infarction (MI) treatment with dual antiplatelet therapy (DAPT). We evaluated the effects of PPIs in reducing the risk of severe UGI bleeding in patients with MI receiving DAPT.

**Methods:**

This retrospective cohort study included patients admitted for acute MI between 2014 and 2018, based on a nationwide health claims database in Korea. Primary outcome was admission for severe UGI bleeding requiring transfusion within 1 year of MI diagnosis. A multivariable Cox regression model was used to calculate the association between PPI use and severe UGI bleeding risk.

**Results:**

Of 100,556 patients with MI on DAPT (mean age, 63.7 years; 75.4% men), 37% were prescribed PPIs. Based on risk assessment for UGI bleeding, among 6,392 (6.4%) high-risk and 94,164 (93.6%) low-risk patients, 50.5% and 35.8% received PPIs, respectively. Overall, 0.5% of the patients experienced severe UGI bleeding within 1 year after MI. The use of PPI was associated with a reduced risk of severe UGI bleeding (hazard ratio [HR], 0.57; 95% confidence interval [CI], 0.47–0.70; *P* < 0.001). The benefits of PPIs were consistent in high-risk (HR, 0.71; 95% CI, 0.45–1.13; *P* = 0.147) and low-risk (HR, 0.54; 95% CI, 0.43–0.68; *P* < 0.001) patients (P for interaction = 0.481).

**Conclusions:**

Among Korean patients with MI receiving DAPT, PPIs were underutilized, even among those at high risk of severe UGI bleeding. Nonetheless, PPI use reduced severe UGI bleeding in low- and high-risk groups.

**Supplementary Information:**

The online version contains supplementary material available at 10.1007/s44197-024-00267-9.

## Introduction

Dual antiplatelet therapy (DAPT) plays a key role in the management of patients with acute myocardial infarction (MI). However, it is associated with an increased risk of bleeding complications [1, 2]. Consequently, recent studies have emphasized the importance of reducing both hemorrhagic and ischemic events with DAPT [3, 4]. Gastrointestinal (GI) bleeding, particularly upper GI (UGI) bleeding, is a significant complication in patients receiving DAPT. Proton pump inhibitors (PPIs) have been shown to reduce the risk of UGI bleeding in these patients [5, 6].

Until now, guidelines have offered conflicting recommendations regarding the prescription of PPIs for patients with MI on DAPT [1, 7–9]. The 2017 European Society of Cardiology (ESC) guidelines recommend prescribing PPIs to all patients with MI, irrespective of their risk profile [7]. In contrast, the 2023 ESC, American, and Korean guidelines recommend using only PPIs in patients at high risk for UGI bleeding [1, 8, 9]. Moreover, only a few studies have examined the real-world usage of PPIs and their effects on UGI bleeding, according to the risk of bleeding [10–13].

In this study, we aimed to investigate the real-world use of PPIs after MI on DAPT, and the comparative effectiveness of PPIs in preventing severe UGI bleeding among these patients by considering guideline-recommended risk groups using a nationwide population-based database.

## Methods

### Data Source and Participants

This retrospective cohort study used data from the Korean National Healthcare Database. Korea operates a universal health insurance system for public health. The National Health Insurance Service (NHIS) covers healthcare costs of all Korean citizens [14]. The Health Insurance Review and Assessment Service (HIRA) is responsible for reviewing all NHIS health claims and quality assessments. In current study, patients diagnosed with MI between January 2014 and December 2018 were selected from the HIRA database. Patients with MI were defined as those admitted with a primary diagnosis code for acute MI (International Classification of Diseases [ICD]-10 code, I21). Considering this criteria for diagnosing acute MI using health claims data in Korea, a validation study in 2013 showed a diagnostic accuracy of 93% [15, 16]. Index date was defined as the date of MI admission (Supplemental Figure S1).

We identified comorbidities and medications of the study participants based on health claims data with ICD-10 diagnosis codes and the Anatomical Therapeutic Chemical Classification System codes (Supplementary Table S1). Using prescription records in the HIRA database, medications of the study participants, including antiplatelet agents, statins, non-steroidal anti-inflammatory drugs (NSAIDs), steroids, H2 blockers/other gastroprotective agents, and proton pump inhibitors (PPIs), were identified and classified. The use of a specific medication was defined as taking a medication for at least 21 days in a 30-day period following acute MI (Supplemental Figure S1). The Korean NHIS only covers DAPT combinations of aspirin and additional antiplatelet agents, such as clopidogrel, ticlopidine, prasugrel, ticagrelor, triflusal, or cilostazol. Therefore, patients receiving DAPT were defined as those treated with aspirin combined with another antiplatelet agent. Dual antiplatelet therapy with a potent P2Y12 inhibitor was additionally defined as the administration of aspirin with prasugrel or ticagrelor. We excluded patients treated with concomitant anticoagulants, those followed up for ≤ 30 days, and those whose admission for MI extended beyond 30 days from the index date. This exclusion was done to eliminate patients with complications during admission and early events associated with poor medical conditions. Also, we aimed to define patients with medications within sufficient observation periods of one month [17], because this study focuses on the role of proton pump inhibitors in patients treated with DAPT, making the precise definition of medication users critical. This definition of medication users inevitably excluded patients with primary outcomes and deaths within one month to ensure equal time for all patients to claim prescriptions for medications and to minimize the risk of immortal time bias. Detailed information based on the claims data is described in Supplemental Methods and Table S1.

### Bleeding Risk Assessment

To identify patients at a high risk for UGI bleeding, we applied the criteria from the 2010 guidelines of the American College of Gastroenterology, American College of Cardiology, and American Heart Association [8]. In the guidelines, the use of PPIs is recommended for patients requiring antiplatelet therapy who have a history of UGI bleeding or multiple risk factors, including advanced age, concurrent use of nonsteroidal anti-inflammatory drugs (NSAIDs), steroids, or anticoagulants, and *Helicobacter pylori* infection [8]. In this study, patients on anticoagulants were excluded, and patients with *Helicobacter pylori* infection could not be precisely identified in the Korean claims-based data. Finally, patients with recent UGI bleeding or those with two of the following risk factors (age ≥ 65 years, or use of NSAIDs or steroids) were defined as having a high risk of UGI bleeding (Supplemental Table S1).

### Outcomes and Follow-ups

Primary outcome was severe UGI bleeding occurring within 1 year post-MI. Severe UGI bleeding was identified on admission with related primary diagnosis of ICD-10 codes and receipt of red blood cell transfusion during admission (Supplemental Table S1). Secondary outcomes included UGI bleeding, which was defined as admission with a related primary diagnostic code without the requirement for transfusion, and all types of GI bleeding, which encompassed admissions with a primary diagnosis of both UGI and lower GI bleeding (Supplemental Table S1). After an index admission for MI, the patients were followed-up until either primary outcome development, loss of NHIS eligibility due to emigration, death, end of the study period (December 31, 2019), or 1 year after the index date, whichever came first.

### Statistical Analyses

Differences between groups were evaluated using the independent t-test, analysis of variance, or Kruskal–Wallis test for continuous variables, and the chi-square test for categorical variables, as appropriate. The Cochran–Armitage test was used to assess the time trends of PPI use among patients with MI during the study period between 2014 and 2018. Cumulative incidence curves for severe UGI bleeding were plotted for those treated with and without PPIs, and a log-rank test was performed. To evaluate the effect of PPIs in reducing the risk of severe UGI bleeding, we used a multivariable Cox regression model and calculated the adjusted hazard ratio (aHR) and 95% confidence interval (CI). Adjustments were made for age, sex, hypertension, diabetes mellitus, chronic kidney disease, liver disease, malignancy, functional dyspepsia, recent upper GI bleeding, and use of statins, NSAIDs, steroids, and H2 blockers/other gastroprotective agents. For secondary outcome analysis, we constructed two individual Cox regression models for UGI bleeding and all types of GI bleeding. To address potential bias from different baseline characteristics between PPI users and non-users, we conducted a sensitivity analysis employing 1:1 propensity score matching (PSM) based samples (Supplemental Methods). Following PSM, stratified Cox regression analyses were performed to evaluate the association between PPI use and the outcomes.

Subgroup analyses were performed according to the bleeding risk defined by the American guideline risk assessment [8]. Additional subgroup analyses were performed to investigate whether the association between PPI use and the risk of severe UGI bleeding differed according to the following factors: sex, age, history of recent UGI bleeding, concomitant use of NSAIDs, H2 blockers/other gastroprotective agents, and potent P2Y12 inhibitors. We also evaluated the risk of severe UGI bleeding, according to the type of PPIs used. Statistical analyses were performed using SAS (version 9.4.2; SAS Institute) and R (version 3.5.1; R Foundation for Statistical Computing) software. Statistical significance was set at *P* < 0.05.

## Results

### Study Population and Baseline Characteristics

**Fig. 1:**
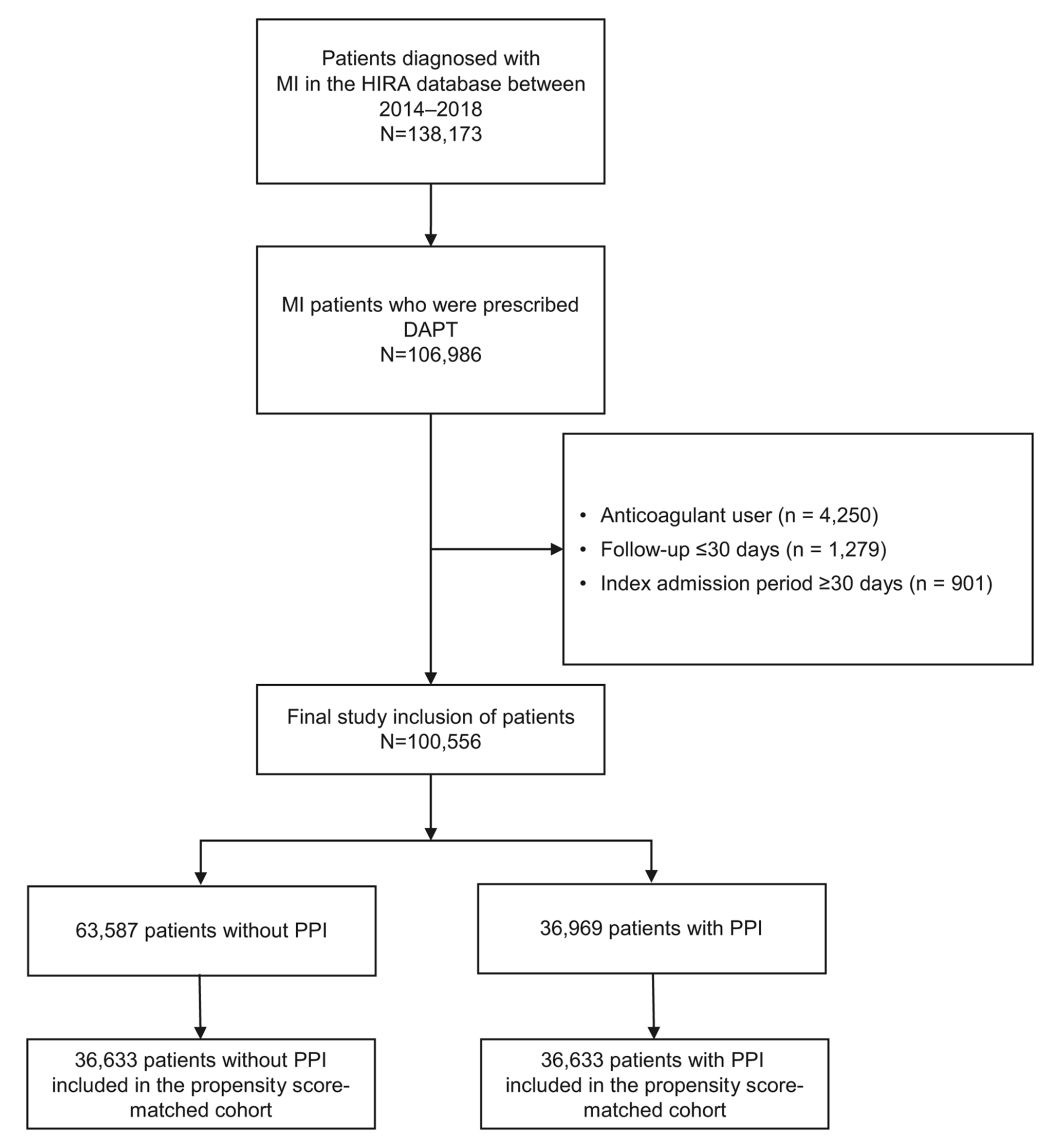
Flow diagram of patient inclusion DAPT: dual-antiplatelet therapy; HIRA: Health Insurance Review and Assessment Service; MI: myocardial infarction; PPI: proton pump inhibitor

Between January 2014 and December 2018, 138,173 patients with acute MI were identified, of whom 106,986 were treated with DAPT (Fig. 1). After applying the exclusion criteria, 100,556 patients with MI treated with DAPT were finally included (mean age ± standard deviation, 63.7 ± 12.8 years; and 75,874 [75.4%] were men). Of these, 36,969 (36.8%) patients were treated with PPIs. Compared with those not using PPI, patients using PPIs were older, more likely to be men, and had a higher prevalence of cardiovascular risk factors, functional dyspepsia, and a recent history of UGI bleeding (Table 1).

**Table 1:** Baseline characteristics of patients with and without proton pump inhibitors before and after propensity score matching

	Before propensity score matching			After propensity score matching		
	Without PPI (*n* = 63,587)	With PPI (*n* = 36,969)	SMD	Without PPI (*n* = 36,633)	With PPI (*n* = 36,633)	SMD
Sex, male	49,489 (77.8)	26,385 (71.4)	-0.143	26,545 (72.5)	26,267 (71.7)	-0.017
Age, years	62.8 ± 12.7	65.1 ± 12.8	0.177	64.6 ± 12.8	65.0 ± 12.8	0.030
Comorbidities						
Hypertension	54,711 (86.0)	32,298 (87.4)	0.040	31,961 (87.3)	31,975 (87.3)	0.001
Diabetes mellitus	21,046 (33.1)	13,037 (35.3)	0.045	12,769 (34.9)	12,932 (35.3)	0.009
Heart failure	29,775 (46.8)	20,180 (54.6)	0.156			
Prior MI	7,104 (11.2)	4,160 (11.3)	0.002			
Chronic kidney disease	4,753 (7.5)	3,453 (9.3)	0.064	3,318 (9.1)	3,367 (9.2)	0.005
COPD	7,727 (12.2)	6,194 (16.8)	0.123			
Liver disease	2,131 (3.4)	1,457 (3.9)	0.030	1,398 (3.8)	1,437 (3.9)	0.006
Cancer	2,691 (4.2)	1,919 (5.2)	0.043	1,837 (5.0)	1,882 (5.1)	0.006
Functional dyspepsia	2,637 (4.2)	1,892 (5.1)	0.044	1,836 (5.0)	1,867 (5.1)	0.004
Recent UGI bleeding	1,462 (2.3)	1,486 (4.0)	0.088	1,242 (3.4)	1,379 (3.8)	0.019
Percutaneous coronary intervention	52,432 (82.5)	29,314 (79.3)	0.088			
Concomitant medications						
Statin	60,284 (94.8)	35,414 (95.8)	0.049			
NSAIDs	1,872 (2.9)	1,824 (4.9)	0.092	1,451 (4.0)	1,627 (4.4)	0.022
Steroids	747 (1.2)	757 (2.1)	0.066	612 (1.7)	680 (1.9)	0.013
H2 blockers/ other gastroprotective agents	16,781 (26.4)	4,596 (12.4)	-0.423	4,519 (12.3)	4,595 (12.5)	0.006

Data are represented as numbers (%) or means ± standard deviation

COPD, chronic obstructive pulmonary disease; MI, myocardial infarction; NSAIDs, non-steroidal anti-inflammatory drugs; PPI, proton pump inhibitor; SMD, standardized mean difference; UGI, upper gastrointestinal

### Primary and Secondary Outcomes

**Fig. 2:**
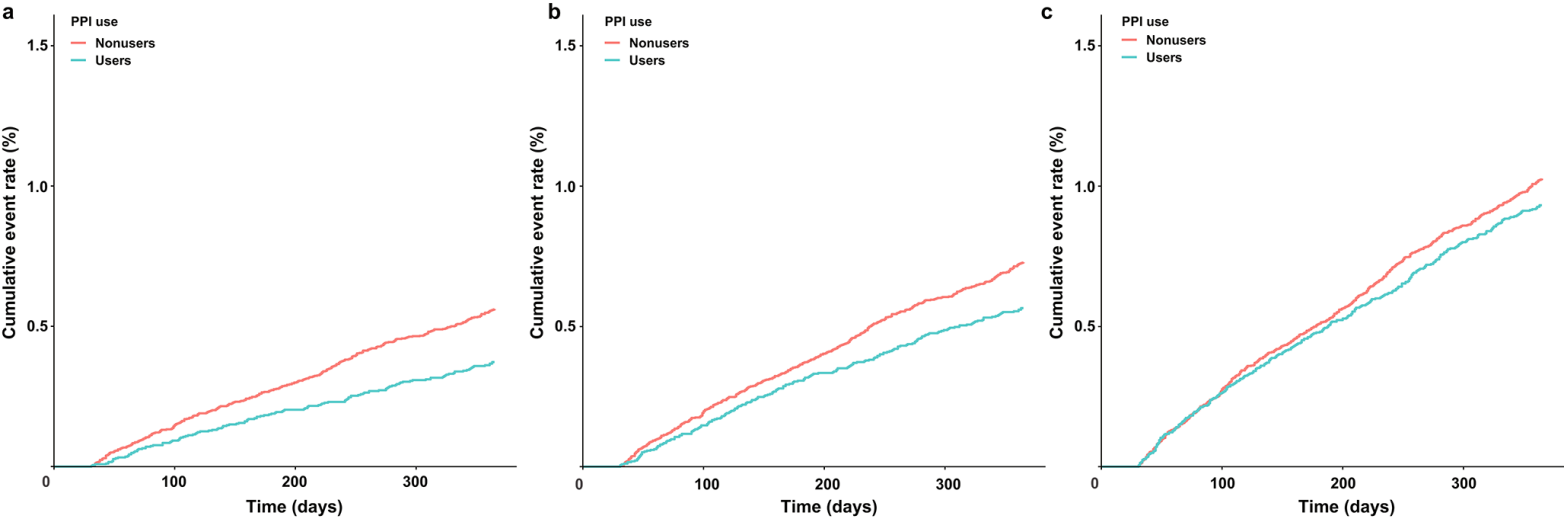
Cumulative incidence of severe UGI bleeding (**a**), UGI bleeding (**b**), and all GI bleeding (**c**) PPI reduced the risk of severe UGI bleeding after MI treatment with DAPT for over a 1-year period (log-rank test, *P* < 0.001, **a**). Regarding the secondary outcome, PPI reduced the risk of UGI bleeding (log-rank test, *P* = 0.002; **b**) and all types of GI bleeding (log-rank test, *P* = 0.100; **c**) GI: gastrointestinal; UGI: upper GI; PPI: proton pump inhibitor; MI: myocardial infarction; DAPT: dual antiplatelet therapy

During the 1-year study period after MI, 487 (0.5%) patients had the primary outcome of severe UGI bleeding. A cumulative incidence curves show a decreased risk of severe UGI bleeding, according to PPI use (*P* < 0.001; Fig. 2a). Regarding the secondary outcomes, 662 (0.7%) patients had UGI bleeding, and 982 (1.0%) had all types of GI bleeding. The use of PPI was associated with a reduced risk of UGI bleeding (*P* = 0.002, Fig. 2a); however, it was not associated with a reduction in all types of GI bleeding (*P* = 0.100, Fig. 2c) after MI.

In the multivariable Cox regression analysis, PPI use was associated with a 43% reduced risk of the primary outcome, severe UGI bleeding (aHR, 0.57; 95% CI, 0.47–0.70; *P* < 0.001; Table 2). The primary outcome was also associated with advanced age, presence of diabetes, chronic kidney disease, recent UGI bleeding, and the use of NSAIDs and steroids (Supplemental Table S2). Regarding the secondary outcomes, the use of PPIs was consistently associated with a decreased risk of UGI bleeding (aHR, 0.68; 95% CI, 0.58–0.81; *P* < 0.001) and all types of GI bleeding (aHR, 0.82; 95% CI, 0.71–0.94; *P* = 0.003; Table 2). Regarding the type of PPI used, the multivariable analysis showed that the associations between PPI use and reduced risk of severe UGI bleeding were consistent across the types of PPIs (P for interaction > 0.999, Supplementary Table S3).

**Table 2:** Effect of proton pump inhibitors on bleeding outcomes after myocardial infarction treatment using dual-antiplatelet therapy

	Severe UGI bleeding			UGI bleeding			All GI bleeding		
	Event	HR (95% CI)	*P* value	Event	HR (95% CI)	*P* value	Event	HR (95% CI)	*P* value
Before propensity score matching^*^	487	0.57 [0.47–0.70]	< 0.001	662	0.68 [0.58–0.81]	< 0.001	982	0.82 [0.71–0.94]	0.003
After propensity score matching†	360	0.59 [0.47–0.73]	< 0.001	497	0.69 [0.57–0.82]	< 0.001	739	0.82 [0.71–0.95]	0.009

^*^Data were obtained using a multivariable Cox proportional hazard regression model for outcome development. Adjustments were made for sex, age, hypertension, diabetes mellitus, heart failure, chronic kidney disease, liver disease, malignancy, functional dyspepsia, recent UGI bleeding, and use of statins, NSAIDs, steroids, and H2 blockers/other gastroprotective agents

†A 1:1 propensity score matching was performed using a logistic regression model for the use of PPI (Supplementary methods) and stratified Cox regression analysis was performed in matched cohort

HR, hazard ratio; CI, confidence interval; GI, gastrointestinal; UGI, upper GI; NSAIDs, non-steroidal anti-inflammatory drugs

### Sensitivity Analysis Using PSM

After a 1:1 PSM, 73,266 patients were included in the matched cohort (36,633 with PPI use, and 36,633 without, Fig. 1). The matched cohort was well balanced in terms of absolute standardized mean difference < 0.1 (Table 1). A stratified Cox proportional hazard regression analysis with selected patients showed that PPI use was consistently associated with a decreased risk of severe UGI bleeding (HR, 0.59; 95% CI, 0.47–0.73; *P* < 0.001). The use of PPI was associated with a decreased risk of UGI bleeding (HR, 0.69; 95% CI, 0.57–0.82; *P* < 0.001), and all types of GI bleeding (HR, 0.82; 95% CI, 0.71–0.95; *P* = 0.009) (Table 2).

### Subgroup Analysis According to Risk Groups

**Fig. 3:**
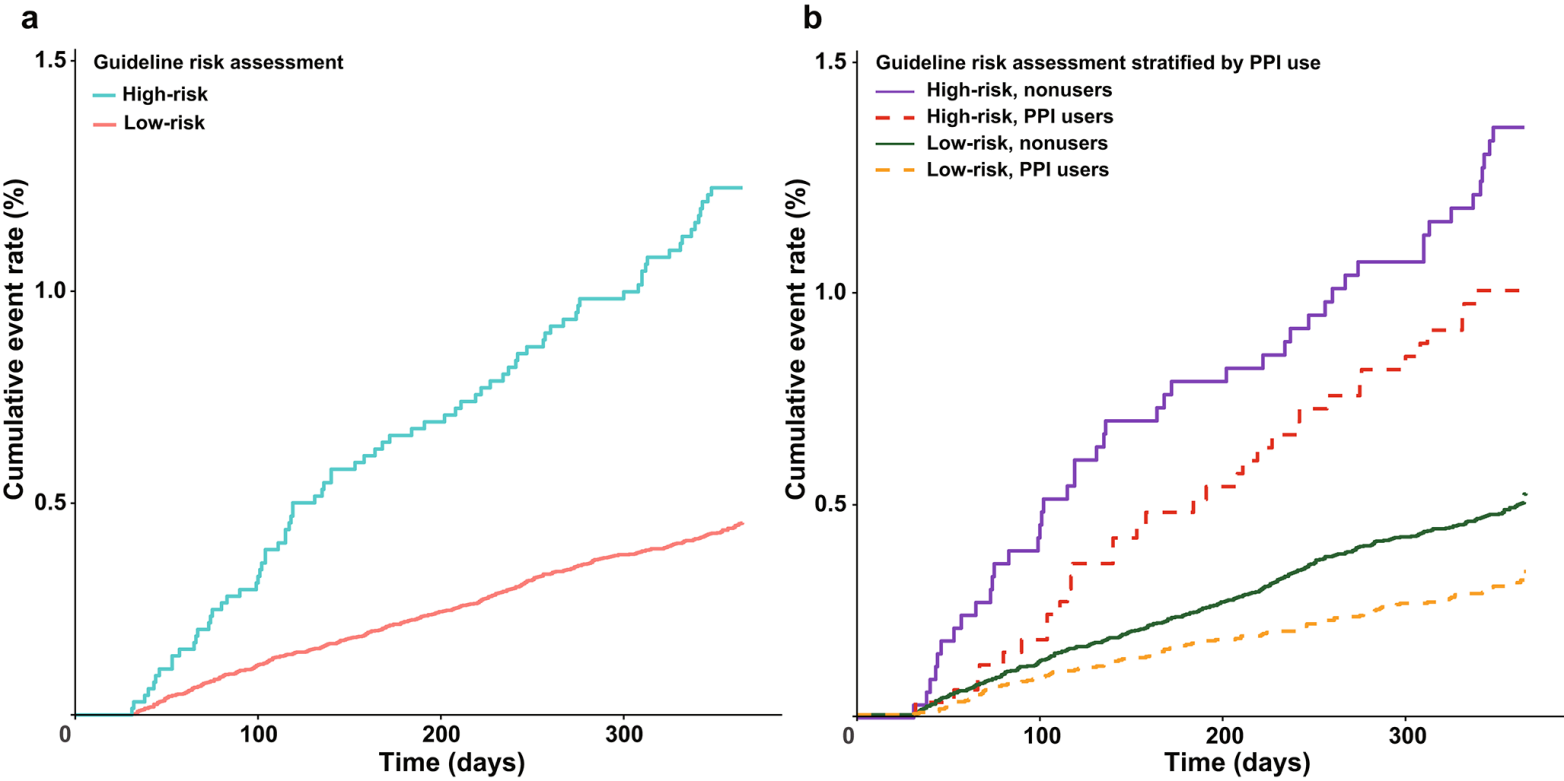
Cumulative incidence of severe UGI bleeding, according to risk groups defined by the American risk assessment (**a**), and stratified by PPI use (**b**). Severe UGI bleeding occurred more commonly in high-risk groups than that in the low-risk groups (log-rank test, *P* < 0.001, **a**), and PPI decreased severe UGI bleeding in both the high- and low-risk groups (log-rank test, *P* < 0.001, **b**) UGI, upper gastrointestinal, PPI, proton pump inhibitor

**Fig. 4:**
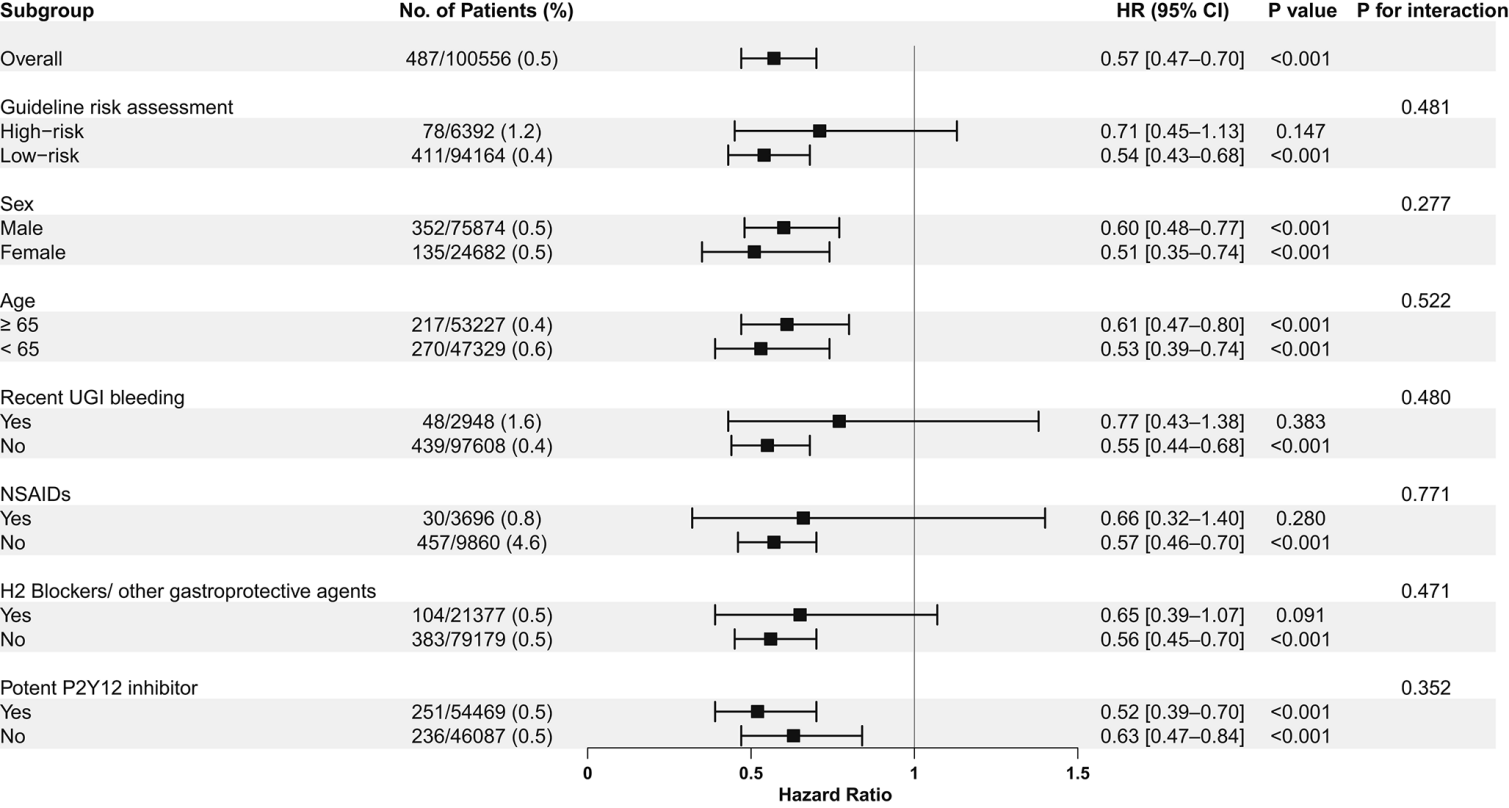
Subgroup analysis of primary outcome CI: confidence interval; HR: hazard ratio; UGI: upper GI; PPI: proton pump; UGI: upper gastrointestinal; NSAIDs: non-steroidal anti-inflammatory drugs

Based on the American guidelines risk assessment, 6,392 (6.4%) patients were classified into a high-risk group for UGI bleeding. The PPIs were prescribed for 3,229 (50.5%) patients in the high-risk group, and 33,740 (35.8%) in the low-risk groups. During the study period between 2014 and 2018, PPI use significantly increased in both the high-risk (41.1–58.1%) and low-risk (25.5–45.6%) groups (all P for trend < 0.001, Supplementary Figure S2). Severe UGI bleeding occurred more commonly in the high-risk group (1.2%) than in the low-risk group (0.4%) (*P* < 0.001; Fig. 3a), and PPI use was associated with a lower risk of severe UGI bleeding in both groups (*P* < 0.001; Fig. 3b). The use of PPIs was associated with a decreased risk of severe UGI bleeding (HR, 0.54; 95% CI, 0.43–0.68; *P* < 0.001) in the low-risk group, and it showed a tendency of decreased risk (HR, 0.71; 95% CI, 0.45–1.13; *P* = 0.147) in the high-risk group (Fig. 4). No significant interaction was observed between PPI use and risk assessment in relation to severe UGI bleeding (*P* = 0.481; Fig. 4).

In the subgroup analyses based on sex, age, history of recent UGI bleeding, and the use of NSAID or H2 blockers/other gastroprotective agents, significant associations between PPI use and a reduced risk of severe UGI bleeding were consistently noted across all the subgroups (Fig. 4). Notably, PPI use was associated with a decreased risk of severe UGI bleeding in both potent P2Y12 inhibitor users (HR, 0.52; 95% CI, 0.39–0.70; *P* < 0.001) and non-users (HR, 0.63; 95% CI, 0.47–0.84; *P* < 0.001) (P for interaction = 0.352, Fig. 4).

## Discussion

Using a nationwide claims database in Korea, we investigated the use of PPIs in relation to the risk of severe UGI bleeding in patients with MI who were treated with DAPT. Although there was a notable increase in the utilization of PPIs during the study period, the PPIs were used in only half of the high-risk patients and one-third of the low-risk patients in Korea. PPI use showed a consistent reduction in the risk of severe UGI bleeding in patients on DAPT after MI, regardless of the assessed UGI bleeding risk or the use of potent P2Y12 inhibitors. These findings suggest that the routine use of PPIs may be reasonable for preventing severe UGI bleeding, a potentially fatal complication, in patients with MI who are treated with DAPT.

PPIs have been recommended to protect against UGI bleeding in patients with MI at increased risk of UGI bleeding when DAPT is required [1, 7–9]. However, our results showed that in Korean real-world practice, PPIs were used in only half of the high-risk groups. There is a paucity of studies examining the real-world use of PPIs in patients on DAPT, or their impact on UGI bleeding, particularly with respect to stratifying bleeding risk [10, 11, 18]. Previous studies have also revealed that PPIs were significantly under-prescribed in high-risk patients with MI on DAPT, with 65–75% of them were not being treated with PPIs [11, 18]. One possible explanation for this low usage could be the concerns over potential adverse effects associated with long-term PPI use, including reduced efficacy of DAPT (particularly with clopidogrel) and heightened risks of kidney disease, bone fractures, infections, cardiovascular events, dementia, and cancer [19]. However, none of these concerns have been conclusively proven in randomized controlled trials. Increased complications with PPIs in observational studies are likely due to residual confounding related to conditions treated with PPIs, rather than a true relationship [20, 21]. The controversy is exemplified by studies utilizing the same Korean HIRA database, where a study found an increased risk of gastric cancer with PPI use, while another did not [22, 23]. Notably, in a large placebo-controlled randomized trial, PPI use over a 3-year period was not linked to any adverse events, except for a possible increase in the risk of enteric infections [24]. Hence, the American Gastroenterology Association’s expert review recommended not only the regular review of the need for PPIs and de-prescribing of PPIs in patients without a definite indication, considering theoretical risks, but also advised against using PPI-associated adverse events as an independent indication for PPI withdrawal [25].

In this study, PPI use decreased the risk of severe UGI bleeding in patients with MI treated with DAPT; this effect remained consistent in the PSM analysis. This benefit remained consistent in the subgroup analyses, regardless of the patients’ risk classification and use of potent P2Y12 inhibitors. According to the Clopidogrel and the Optimization of Gastrointestinal Events Trial (COGENT), which is the only double-blind randomized trial evaluating the efficacy and safety outcomes of concomitant administration of clopidogrel-based DAPT and PPIs in patients with coronary artery disease, prophylactic use of PPIs reduced the rates of all GI bleeding by 34% and overt UGI bleeding by 13% [5]. Additionally, another randomized trial showed a numerical reduction in the incidence of UGI bleeding when an extended risk assessment for bleeding was routinely performed; however, this study could not demonstrate any significant effect due to lack of statistical power [10]. There have been differences in guidance regarding PPI use in patients with MI on DAPT [1, 7–9], which may be attributed to variations in the interpretation of these trials [5, 10]. The 2017 ESC guidelines regarding DAPT use in coronary artery disease recommended routine use of PPI, regardless of risk score [7]. In contrast, the 2023 ESC guidelines for the management of acute coronary syndromes recommended the use of PPIs for patients receiving any antithrombotic regimen who are at high risk of GI bleeding [1]. Both the 2010 American and 2020 Korean guidelines also emphasized that patients on DAPT with multiple risk factors for GI bleeding can be appropriately prescribed PPIs [8, 9]. Our study suggested that PPI use may decrease the risk of severe UGI bleeding, even in low-risk groups. However, future randomized trials are required to address this issue.

In another interpretation, our study results suggest the need for better risk stratification for severe UGI bleeding [11]. Although the American guidelines defined high-risk group as having a significantly higher 1-year risk for severe UGI bleeding compared to the low-risk group, we found that < 20% of UGI bleeding actually occurred in this high-risk group. Furthermore, this study showed that PPI use was associated with a 29% reduction in severe UGI bleeding in the high-risk group, and a 46% reduction in the low-risk group. The greater benefits of PPIs in the low-risk group underscore the challenge of pinpointing the optimal candidates for PPI therapy. These results may be attributable to the small number of patients in the high-risk group. However, in a previous European study, which used the ESC guideline-based risk stratification, it also showed that PPI use was associated with a 24% reduction in UGI bleeding in the high-risk group, and a 51% reduction in the low-risk group [11]. Concisely, our findings suggest that a more accurate alternative assessment is needed to better identify high-risk groups that would benefit from PPI use in preventing UGI bleeding. The Academic Research Consortium for High Bleeding Risk recently announced a new consensus definition for patients with high bleeding risk after coronary intervention [4]. However, applying this bleeding risk assessment in routine clinical practice or research using claims databases may be challenging, as 20 clinical criteria need to be considered [4]. These results suggest that in real-world practice, identifying patients who would benefit from PPIs is not straightforward, and rather than stratifying bleeding risk, active consideration should be given to the use of PPIs in patients who are on DAPT.

This study has several limitations. First, because the outcomes were assessed only after 30 days from admission, early and in-hospital outcomes were not considered. Second, we defined PPI users as time-invariant variable according to their usage pattern within 30 days after the index date. Due to the complexity of defining medication use as a time-dependent variable, many previous studies have determined medication use as a time-invariant binary variable based on exposure for a predefined period and investigated the relationship between medication use and long-term outcomes (landmark analysis) [26, 27]. We defined patients with medications during sufficient observation periods (1 month) [17]. Third, certain risk factors for GI bleeding, such as *Helicobacter pylori* infection, history of gastroesophageal reflux disease, and chronic alcohol use, were not available in the health claim database, limiting our ability to identify all high-risk patients. Fourth, suboptimal compliance with PPI use may have influenced the estimates; however, this could reflect real-world settings, where compliance may not always be optimal. Despite these limitations, this study has several strengths. First, we included a substantial number of patients with MI receiving DAPT using a nationwide health claims database. The sample size was larger than that of prior claims-based study [11]. Second, a major advantage of this study is its nationwide scope, which minimizes the risk of selection bias.

In conclusion, among Korean patients with acute MI receiving DAPT, PPI use was associated with a decreased 1-year risk of severe UGI bleeding in the entire population and across all risk groups. Proton pump inhibitor use increased during the study period, but remained underutilized, as only half of the high-risk group patients were treated with PPIs, according to the American guideline risk assessment. Therefore, an improved risk stratification system is required to screen high-risk groups, and active adherence to PPI guidelines should be encouraged.

## Electronic Supplementary Material

Below is the link to the electronic supplementary material.

Supplementary Material 1

## Data Availability

Researchers can access the HIRA database by submitting requests to the Korean Health Insurance Review Health Big Data Hub (https://opendata.hira.or.kr).
