# Supplementary material for: Proton Pump Inhibitor for Gastrointestinal Bleeding in Patients with Myocardial Infarction on Dual-Antiplatelet Therapy: A Nationwide Cohort Study
Source: J Epidemiol Glob Health. 2024 Jun 24;14(3):1142–51. doi: 10.1007/s44197-024-00267-9 (PMC11442791; doi:10.1007/s44197-024-00267-9)
Supplement: Supplementary file 1 — Supplementary Material 1 [file 44197_2024_267_MOESM1_ESM.docx]

Supplemental Material

**Proton Pump Inhibitor for Gastrointestinal Bleeding in Patients with Myocardial Infarction on Dual-Antiplatelet Therapy: A Nationwide Cohort Study**

Minyoul Baik, MD^1^; Jimin Jeon, MS^1^; Jinkwon Kim, MD, PhD^1^; Joonsang Yoo, MD^1^

^1^Department of Neurology, Yongin Severance Hospital, Yonsei University College of Medicine, Yongin-si, Gyeonggi-do, South Korea

**Supplemental methods**

We identified comorbidities, including hypertension, diabetes mellitus, heart failure, prior myocardial infarction (MI), chronic kidney disease, chronic obstructive pulmonary disease, liver disease, malignancy, functional dyspepsia, and recent upper gastrointestinal (UGI) bleeding, based on health claims data with International Classification of Diseases (ICD)-10 diagnosis codes (Supplementary Table S1).

To reduce the potential confounding effects of different baseline characteristics, a 1:1 propensity score matching (PSM) analysis was performed. The propensity score was estimated using a logistic regression model for the use of PPI, which included using age, sex, hypertension, diabetes mellitus, chronic kidney disease, liver disease, malignancy, functional dyspepsia, recent upper gastrointestinal (GI) bleeding, and the use of statins, NSAIDs, steroids, H2 blockers, or other gastroprotective agents. We created a 1:1 propensity score-matched cohort by matching each patient who used PPIs with those who did not. A nearest-neighbor-matching algorithm was applied based on a difference of 0.1 times the standard deviation of the logit-transformed propensity scores. Covariate balance was evaluated using standardized mean differences, with a standardized difference of <0.1 considered adequate balance.

**Supplemental Figure S1.** Schematic timelines of the study

**
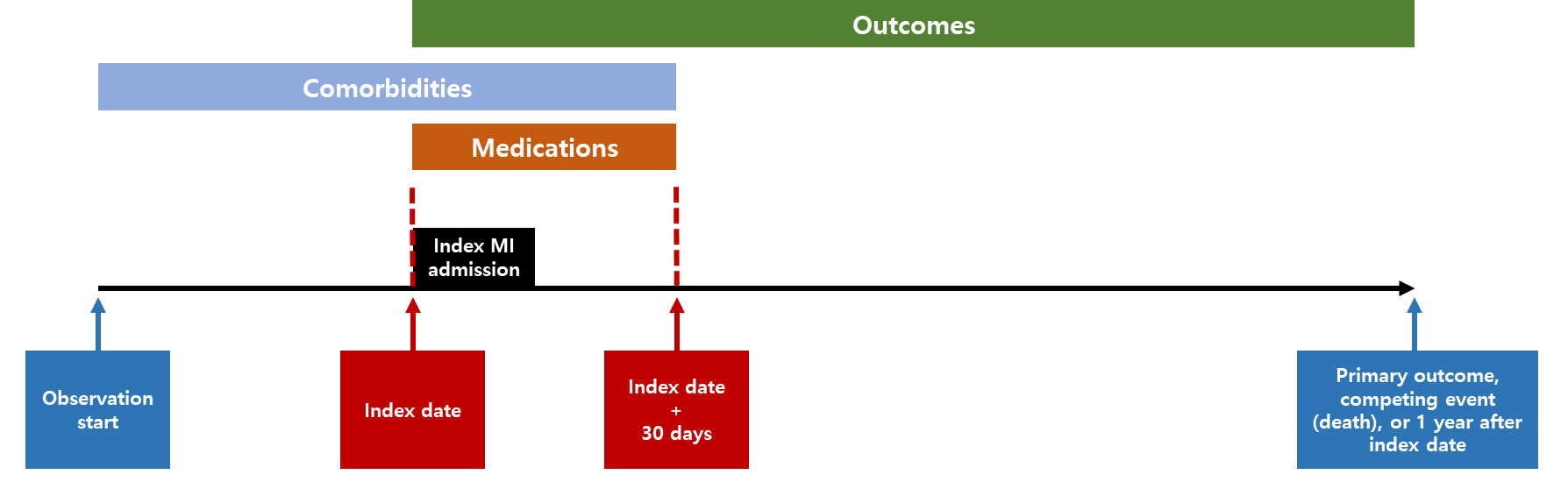
**

MI, myocardial infarction.

**Supplemental Figure S2**. Temporal trends of proton pump inhibitor use stratified by the American Heart Association guideline defined risk groups.
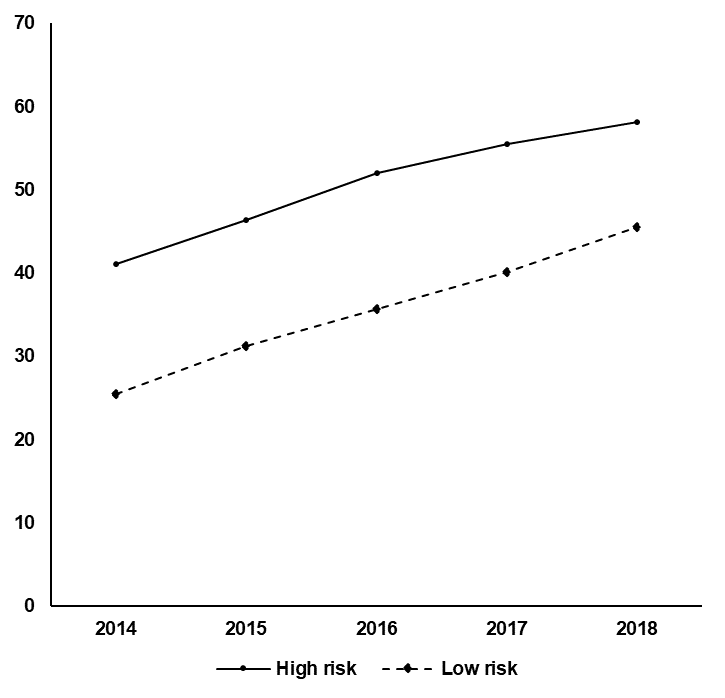


|  | 2014 | 2015 | 2016 | 2017 | 2018 |
| --- | --- | --- | --- | --- | --- |
| High-risk (n=6,392) | 584 (41.1) | 566 (46.4) | 660 (52.6) | 694 (55.5) | 725 (58.1) |
| Low-risk (n=94,164) | 4,689 (25.5) | 5,549 (31.2) | 6,698 (35.7) | 7,788 (40.1) | 9,016(45.6) |

Number (%) of patients using proton pump inhibitors calssified as high and low risk by American guideline risk assessment. P for trend for proton pump inhibitor use during study period in both high-risk and low-risk < 0.05.

**Supplemental Table S1.** Definition of variables based on health claim data

|  | **ICD-10 and claim codes** |
| --- | --- |
| **Inclusion/exclusion criteria** |  |
| Acute myocardial infarction | I21 |
| **Comorbidities** |  |
| Hypertension | I10–I13, I15; and prescription of antihypertensive drug |
| Diabetes mellitus | E10-E14; and prescription of antidiabetic drugs |
| Heart failure | I11.0, I42, I50, I97.1 |
| Prior myocardial infarction | I21–23, I25.2 |
| Prior stroke | I60–64, I69 |
| Chronic kidney disease | N18–19 |
| Liver disease | C22, K70.2, K70.3, K70.4, K74.6, K70.1, B18.0–2 at least 2 times |
| Chronic obstructive pulmonary disease | J42–J44, except J43.0 at least 2 times |
| Malignancy | C00–97; and cancer registration code (V027, V193–4) |
| Functional dyspepsia | K30 |
| Recent UGI bleeding | K22.6, K25.0, K25.2. K25.4, K25.6, K26.0, K26.2, K26.4, K26.6, K27.0, K27.2, K27.4, K27.6, K28.0, K28.2, K28.4, K28.6, K29.0, K92.0, K92.1, I85.0, I98.3, K22.11, K31.81 |
| Percutaneous coronary intervention | M6551–2, M6561–4, M6571–2 |
| Statin | ATC: C10A (atorvastatin, Fluvastatin, lovastatin, pitavastatin, pravastatin, rosuvastatin, simvastatin) |
| NSAIDs | ATC: M01A (but not M01AX05) |
| Steroids | ATC: H02AB |
| H2 blockers/ other gastroprotective agents | Cimetidine (A02BA01), Ranitidine (A02BA02), Famotidine (A02BA03), Nizatidine (A02BA04), Roxatidine (A02BA06), Ranitidine bismuth citrate (A02BA07), Lafutidine (A02BA08), Rebamipide (A02BX14), Misoprostol (A02BB01) |
| Proton pump inhibitors | esomeprazole, pantoprazole, lansoprazole, rabeprazole, omeprazole, ilaprazole, dexlansoprazole |
| **High-risk for UGI bleeding** | 1) Recent UGI bleeding  or  2) Two of followings  A. Age ≥65  B. NSAIDs  C. Steroid |
| **Outcome** |  |
| Severe UGI bleeding | Admission with the primary diagnosis of K22.6, K25.0, K25.2. K25.4, K25.6, K26.0, K26.2, K26.4, K26.6, K27.0, K27.2, K27.4, K27.6, K28.0, K28.2, K28.4, K28.6, K29.0, K92.0, K92.1, I85.0, I98.3, K22.11, K31.81 and claims of red blood cell transfusion (X2021, X2022, X2031, X2032, X2131, X2132, X2091, X2092, X2111, X2112, X2515, X2512) |
| UGI bleeding | K22.6, K25.0, K25.2. K25.4, K25.6, K26.0, K26.2, K26.4, K26.6, K27.0, K27.2, K27.4, K27.6, K28.0, K28.2, K28.4, K28.6, K29.0, K92.0, K92.1, I85.0, I98.3, K22.11, K31.81 |
| All types of GI bleeding | K22.6, K25.0, K25.2, K25.4, K25.6, K26.0, K26.2, K26.4, K26.6, K27.0, K27.2, K27.4, K27.6, K28.0, K28.2, K28.4, K28.6, K29.0, K62.5, K66.1, K92.0, K92.1, K92.2, I85.0, I98.3, K62.5, K22.11, K31.81 |

Comorbidities were generally defined from the earliest possible date in the data to the index date +30 days, except for the following variables. Prior myocardial infarction was defined from the earliest possible date in the data to the index date -1 day. Recent UGI bleeding was defined from the index date -1 year to the index date +30 days. Percutaneous coronary intervention was defined from the index date to the index date +30 days. The use of a specific medication was defined as taking the medication for at least 21 days in a 30-day period following index date. The schematic timeline is provided in Supplemental Figure S1.

ATC, Anatomical Therapeutic Chemical Classification System; ICD, International Classification of Diseases; GI, gastrointestinal; UGI, upper GI; NSAIDs, nonsteroidal anti-inflammatory drugs.

**Supplemental Table S2.** Univariable and multivariable Cox regression for primary outcome

| Variable | Crude HR [95%CI] | Adjusted HR [95%CI] |
| --- | --- | --- |
|  |  |  |
| Sex, male | 0.84 [0.69–1.02] | 1.07 [0.86–1.32] |
| Age, years | 1.02 [1.01–1.03] | 1.02 [1.01–1.03] |
| Comorbidity |  |  |
| Hypertension | 1.19 [0.90–1.58] | 0.98 [0.73-1.30] |
| Diabetes mellitus | 1.73 [1.45–2.06] | 1.47 [1.22-1.77] |
| Heart failure | 1.15 [0.96–1.37] | 1.01 [0.84-1.21] |
| Chronic kidney disease | 2.54 [2.01-3.21] | 1.87 [1.46-2.40] |
| Liver disease | 1.54 [1.04-2.29] | 1.37 [0.92-2.05] |
| All type of cancer | 1.58 [1.11-2.25] | 1.23 [0.86-1.76] |
| Functional dyspepsia | 1.11 [0.73-1.67] | 1.00 [0.66-1.51] |
| Recent UGI bleeding | 3.70 [2.75-4.99] | 3.38 [2.50-4.57] |
| Percutaneous coronary intervention | 1.18 [0.92-1.50] | 1.16 [0.91-1.47] |
| Concomitant medication |  |  |
| Statin | 0.75 [0.52-1.08] | 0.93 [0.64-1.35] |
| NSAIDs | 1.74 [1.20-2.51] | 1.62 [1.10-2.38] |
| Steroids | 2.17 [1.30-3.62] | 1.69 [1.00-2.87] |
| H2 blocker/other gastroprotective agents | 1.01 [0.81-1.25] | 0.82 [0.65-1.02] |
| Proton pump inhibitors | 0.66 [0.54-0.81] | 0.57 [0.47-0.70] |

CI, confidence interval; HR, hazard ratio; NSAIDs, non-steroidal anti-inflammatory drugs; UGI, upper gastrointestinal.

**Supplemental Table S3.** Primary outcome according to PPI type

|  | Number (%) | aHR [95% CI] | P–value |
| --- | --- | --- | --- |
| No PPI | 63,587 (63.2) | Ref |  |
| PPI type^*^ | 36,969 (36.8) |  |  |
| Esomeprazole | 7,872 (7.8) | 0.68 [0.48–0.97] | 0.034 |
| Pantoprazole | 9,137 (9.1) | 0.74 [0.54–1.01] | 0.060 |
| Lansoprazole | 10,238 (10.2) | 0.40 [0.27–0.59] | <.001 |
| Rabeprazole | 4,690 (4.7) | 0.62 [0.38–0.99] | 0.047 |
| Omeprazole | 414 (0.4) | 0.69 [0.17–2.77] | 0.600 |
| Ilaprazole | 397 (0.4) | 0.75 [0.19–2.99] | 0.679 |
| Dexlansoprazole | 4,221 (4.2) | 0.32 [0.16–0.65] | 0.002 |

^*^P for interaction of PPI type > 0.999.

CI, confidence interval; HR, hazard ratio; PPI, proton pump inhibitor.
